# Soil test crop response nutrient prescription equations for improving soil health and yield sustainability—a long-term study under Alfisols of southern India

**DOI:** 10.3389/fpls.2024.1439523

**Published:** 2024-11-13

**Authors:** R. Krishna Murthy, Bhavya Nagaraju, K. Govinda, S. N. Uday Kumar, P. K. Basavaraja, H. M. Saqeebulla, G. V. Gangamrutha, Sanjay Srivastava, Pradip Dey

**Affiliations:** ^1^ All India Coordinated Research Project on Soil Test Crop Response, University of Agricultural Sciences, Bangalore, India; ^2^ All India Coordinated Research Project on Soil Test Crop Response, Indian Council of Agricultural Research (ICAR)-Indian Institute of Soil Science, Bhopal, India; ^3^ Soil Science, Indian Council of Agricultural Research-Agricultural Technology Application Research Institute, Kolkata, India

**Keywords:** STCR, yield, nutrient use efficiency, soil quality, principle component analysis

## Abstract

**Introduction:**

Enhancing soil health and nutrient levels through fertilizers boosts agricultural productivity and global food security. However, careful fertilizer use is essential to prevent environmental damage and improve crop yields. The soil test crop response (STCR) is a scientific approach to fertilizer recommendation that ensures efficient use, supporting higher crop production while protecting the environment and preserving resources.

**Methodology:**

A long-term field experiment on the STCR approach was initiated in 2017 at the Zonal Agriculture Research Station, University of Agricultural Sciences, Bangalore, India. The experiment aimed to study the impact of STCR-based nutrient prescription along with farmyard manure (FYM) for a targeted yield of soybean (*Glycine max*), sunflower (*Helianthus annuus*), dry chili (*Capsicum annuum*), aerobic rice (*Oryza sativa* L.), foxtail millet (*Setaria italica*), okra (*Abelmoschus esculentus*), and kodo millet (*Paspalum scrobiculatum*) on yield and changes in soil health in comparison with other approaches of fertilizer recommendation.

**Results:**

The results showed a significant and positive impact of the integrated use of fertilizer with FYM based on the STCR approach on the productivity of all the crops and soil fertility. Significantly higher yields of soybean (23.91 q ha^−1^), sunflower (27.13 q ha^−1^), dry chili (16.67 q ha^−1^), aerobic rice (65.46 q ha^−1^), foxtail millet (14.07 q ha^−1^), okra (26.82 t ha^−1^), and kodo millet (17.10 q ha^−1^) were observed in the STCR NPK + FYM approach at yield level 1 compared to the general recommended dose and soil fertility rating approach. This approach outperformed the standard recommendations, enhancing nutrient uptake and efficiency across various crops. Utilizing the principal component analysis, the soil quality index effectively reflected the impact of nutrient management on soil properties, with the STCR NPK + FYM treatment at yield level 1 showing the highest correlation with improved soil physical and chemical parameters.

**Discussion:**

The STCR approach led to improved yield, nutrient uptake, utilization efficiency, and soil health, thanks to a balanced fertilization strategy. This strategy was informed by soil tests and included factors like crop-induced nutrient depletion, baseline soil fertility, the efficiency of inherent and added nutrients through fertilizers and farmyard manure, and the success of yield-targeting techniques in meeting the nutritional needs of crops.

## 1 Introduction

India’s quest for agricultural abundance in the 21st century hinges on its ability to sustainably satisfy the escalating demands for food, feed, fiber, and fuel amid its burgeoning population (Gulati et al., 2023). The nation has witnessed a dramatic escalation in chemical fertilizer usage, skyrocketing from a mere 69.8 thousand tons in 1950–1951 to a staggering 29.796 million tons in 2021–2022, propelling food grain production from 50.85 to 305.6 million tons (FAI, 2022). However, this reliance on chemical fertilizers, accounting for half of the food grain yield boost (Shukla et al., 2022), has precipitated a cascade of ecological quandaries—from nitrate contamination and soil acidification to eutrophication and greenhouse gas emissions—threatening the very fabric of India’s agricultural sustainability (Kumar et al., 2022).

In the dynamic arena of soil science, the strategic application of plant nutrients emerges as a cornerstone for the enduring yield of crops. Precision in nutrient delivery, tailored to the soil’s inherent nutrient profile and expected crop uptake, is paramount. Yet, the relentless use of potent fertilizers has led to the depletion of essential micro and secondary nutrients, undermining crop yields across various regions (Singh, 2010). The plateau in India’s crop production is largely attributed to outdated fertilizer practices, suboptimal utilization, and disproportionate fertilizer application. A methodical assessment of fertilizer quantities can be instrumental in bolstering yields while concurrently elevating nutrient efficiency. This challenge is met by adopting an integrated nutrient management approach, combining organic, biological, and synthetic fertilizers. It is acknowledged that neither organic manures nor chemical fertilizers alone can fulfill the quest for food and nutritional security (Paramesh et al., 2023). The habitual incorporation of organic matter not only invigorates soil life and diversity but also ameliorates its physical structure (Kumar and Tripathi, 2009). Thus, the synergistic merger of inorganic and organic inputs is pivotal in perpetuating crop productivity and augmenting soil vitality, as evidenced by their mutual benefits (Antil et al., 2011).

On the other hand, the art of balanced fertilization involves the precise deployment of vital plant nutrients, tailored in perfect harmony and quantity to meet the unique demands of each crop scenario. The innovative approach of calibrating nutrient recommendations through the soil test crop response (STCR) for targeted yield using developed fertilizer adjustment equations offers a superior strategy for the judicious application of nutrients (Sharma et al., 2016). Such meticulous fertilizer guidance is rendered with prudence, taking into account yield dynamics and desired agronomic efficiencies while also acknowledging the nutrient contributions from both external sources, such as chemical and organic fertilizers, and internal, indigenous soil reserves (Haokip et al., 2023). The STCR approach is used to determine the optimal fertilizer recommendations for crops based on soil test values. This approach helps in achieving the yield target of a crop while maintaining soil fertility and minimizing environmental impact. Set against this scientific canvas, a pioneering study was embarked upon to evaluate the long-term effects of strategic nutrient management, grounded in soil testing and crop response, on the enduring productivity and vitality of soil within a *Typic Haplustalf* setting.

## 2 Materials and methods

### 2.1 Experimental site

A field experiment has been conducted since 2017 at the Zonal Agricultural Research Station, University of Agricultural Sciences, Bangalore, Karnataka, India. This site is characterized by a dry tropical savanna climate, with hot summers and cool winters, located at 13°04′55.2′′N, 77°34′10.0′′E, and an elevation of 930 m. The well-drained red soil in the study location belonged to the taxonomically defined large group, Typic *Kandic Paleustalfs* belonging to the fine mixed *Isohyperthermic* family. Prior to the experiment, soil samples were taken from the top 15 cm, shade-dried, and analyzed for their physical, chemical, and biological properties. Measurements included a bulk density of 1.39 g/cm³, 35.98% porosity, and 30.10% maximum water holding capacity. Soil chemistry revealed a pH of 5.49, electrical conductivity of 0.09 dS m^−1^, organic carbon content of 0.28%, and levels of available nitrogen, phosphorus, and potassium at 230.50 kg ha^–1^, 58.66 kg ha^–1^, and 120.00 kg ha^–1^, respectively. Biological assessments showed microbial biomass carbon at 115.56 mg kg^–1^, microbial biomass nitrogen at 13.48 mg kg^–1^, and enzyme activities of dehydrogenase, alkaline phosphatase, and acid phosphatase at 35.42 µg TPF g^–1^ 24 h^–1^, 2.01 µg PNP g^–1^ h^–1^, and 5.31 µg PNP g^–1^ h^–1^, respectively.

**Figure 1:**
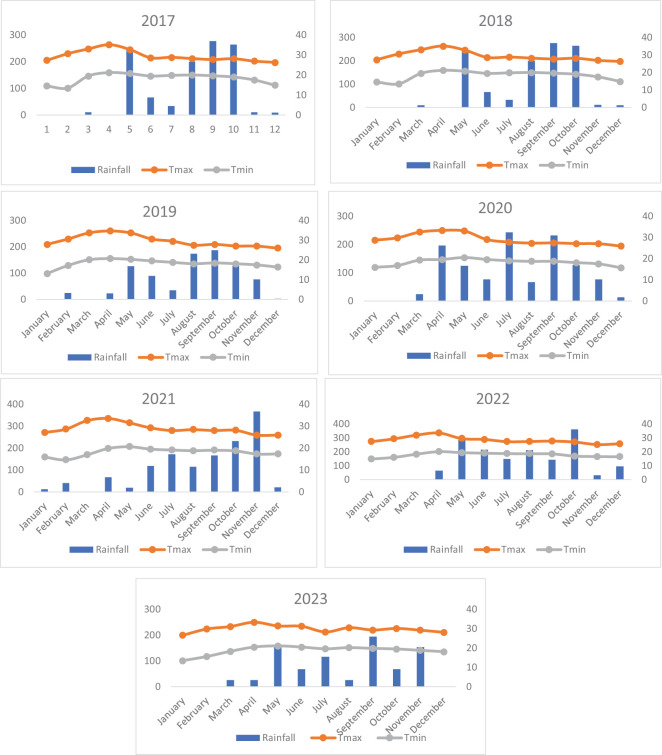
Variation in rainfall and temperature (maximum and minimum) during the experimental period from 2017 to 2023.

**Figure 1** encapsulates a dataset of weather parameters recorded over 6 years, from 2017 to 2022. The data indicate both the highest and average precipitation levels (in millimeters) and temperatures (in degrees Celsius) for each year. From June to September, the weather data reveal distinct patterns in rainfall and temperature. During the crop growth period, rainfall levels in June were moderate, showing a noticeable increase compared to May. July experiences peak rainfall, with the highest levels observed across all years. In August, rainfall remains high but slightly lower than in July, and by September, it starts to decrease, marking the end of the monsoon season. The highest temperatures were recorded in the warmer months, particularly May, with a slight fluctuation but generally close to 35°C. The average temperatures remained relatively stable, with a slight decrease, hovering approximately 20°C to 25°C. The year-to-year variations show some differences in the exact timing and intensity of these peaks, but the overall trend remains consistent.

### 2.2 Experimental design and treatments

The soil test crop response-targeted yield nutrient prescription equations for sunflower, dry chili, aerobic rice, foxtail millet, okra, and kodo millet were developed after developing fertility gradient for each crop by following the guidelines given by Ramamoorthy et al. (1967) in 2016, 2017, 2018, 2019, 2020, 2021, and 2022, respectively, and the STCR-targeted yield nutrient prescription equations for each crop are detailed in **Supplementary Material S1**. Validation of the developed nutrient prescription equations for different crops was conducted in the permanent plot during 2017, 2018, 2019, 2020, 2021, 2022, and 2023. The study was first initiated in 2017, focusing on a soybean crop, and continued annually with different crops. A randomized complete block design was employed, featuring six fertilization treatments plus an absolute control, each replicated thrice. Sequentially, sunflower, dry chili, aerobic rice, foxtail millet, okra, and kodo millet were cultivated from 2018 to 2023. The treatments included T_1_: STCR NPK for yield level 1, T_2_: STCR NPK with farmyard manure (FYM) for yield level 1, T_3_: STCR NPK for yield level 2, T_4_: STCR NPK with FYM for yield level 2, T_5_: general recommended dose, T_6_: soil fertility rating, and T_7_: absolute control. The targeted yield levels 1 and 2 were identified based on the ±20% of the genetic potential yield of the crops. Soil samples from 0 to 15 cm depth were collected before sowing and after the harvest of each crop and analyzed for available N, P, and K. Nitrogen, phosphorus, and potassium sources were urea, single superphosphate, and muriate of potash, respectively. The initial soil test readings and the amount of fertilizer added for each crop are presented in **Supplementary Table S2**. FYM was applied 15 days before sowing; half the nitrogen and full amounts of phosphorus and potassium were applied as basal. For all the crops, standard agronomic practices under irrigated conditions were followed for crop cultivation, and plants were harvested at peak maturity. The economics in terms of value cost ratio (VCR) were computed by using the standard formulae as shown below (Ramamoorthy et al., 1967).


$$\text{VCR}=\frac{\left[\text{Yield in treated plot }\left(\text{kg }{\text{ha}}^{-1}\right)-\text{Yield}\;\text{in}\;\text{treated}\;\text{plot}\;\left(\text{kg }{\text{ha}}^{-1}\right)\right]}{\text{Cost of fertelizers and FYM applied to treated plot}}\times \text{Cost}\;kg^{-1}$$

### 2.3 Analysis of soil and plant samples

The initial and post-harvest soil samples collected from the experimental plot at 0–20 cm depth as per the layout of the experiment after each crop were air-dried under a shade and ground to pass through a 2-mm sieve. Bulk density was determined in the experimental field by using rings of known volume (5 cm inner diameter and 5 cm height). Soil cores were dried at 105°C in an oven for 48 h. Bulk density was calculated by dividing the weight of dried soil by the volume of the core used (Veihmeyer and Hendrickson, 1948). The soil pH was measured in a 1:2.5 soil:water suspension after stirring for 30 min by the potentiometric method using a glass electrode, and electrical conductivity was measured in a supernatant liquid of soil:water (1:2.5) suspension with the help of a conductivity meter as described by Jackson (1973). The organic carbon in soil samples was determined using K_2_Cr_2_O_7_ as an oxidizing agent (1 N) and back titrating with 0.5 N FAS method as suggested by Walkley and Black (1934). The available N was estimated by the alkaline KMnO_4_ method where organic matter present in the soil was oxidized with a hot alkaline KMnO_4_ solution in the presence of NaOH. The ammonia (NH_3_) that evolved during oxidation was distilled and trapped in a boric acid mixed indicator solution. The amount of ammonia trapped was estimated by titrating with standard acid (Subbiah and Asija, 1956), and the available P was extracted with Bray’s extractant (i.e., 0.025 M of HCl and 0.03 M of NH_4_F) and was determined colorimetrically by the ascorbic acid method. The intensity of the blue color was read at 660 nm using a spectrophotometer (Bray and Kurtz, 1945), and the available K was extracted with 1 N of ammonium acetate (pH 7.0) and fed directly to a flame photometer (Page et al., 1982).

Plant samples were collected at the harvesting stage. Five plants from each plot which were randomly selected and labeled were collected by pulling out the entire plant carefully. All the plant samples were washed first with tap water and then with distilled water to remove the adhering soil and dusts. Then, they were air-dried and later dried in a hot air oven at 65°C. The plant samples were ground in a willey mill. The N content in the plant samples was determined by the micro Kjeldahl method using a digestion mixture consisting of copper sulfate, potassium sulfate, and selenium catalytic mixture. Plant samples weighing 1 g were digested in digestion flasks in a macro Kjeldahl unit using sulfuric acid and the digestion mixture. After complete digestion, the digested materials were distilled in an alkaline medium, and the liberated ammonia was trapped in a 4% boric acid solution containing a mixed indicator. The trapped ammonia was titrated against standard sulfuric acid (Piper, 1966). Di-acid extract was prepared as per the method outlined by Jackson (1973). It was carried out using a 9:4 mixture of HNO_3_:HClO_4_. The predigestion of the sample was done by using 10 mL of HNO_3_ g^−1^ sample. This di-acid extract was used to determine P and K content in the plant samples. Phosphorus content in the digested plant sample was estimated by the vanadomolybdophosphoric yellow color method in nitric acid medium, and the color intensity was measured at 460 nm wavelength as described by Jackson (1973) from the di-acid extract. Potassium was estimated from the di-acid extract by atomizing the diluted acid extract in a flame photometer (Jackson, 1973). From the chemical analytical data, the uptake of each nutrient was calculated.


$$\text{Nutrient uptake }\left(\text{kg }{\text{ha}}^{-1}\right)=\text{ Nutrient control }(\%)\times \text{ dry weight }{\text{in kg ha}}^{-1}/100$$

### 2.4 Microbial analysis of the soil

The soil microbial properties, viz., microbial biomass carbon (MBC), microbial biomass nitrogen (MBN), and soil enzymatic activities, were analyzed to know the long-term effect of the integrated use of NPK and FYM under STCR approaches on soil biological properties under a validation trial. The fresh soil samples were immediately transported to the laboratory and stored in a freezer for the assessment of biological parameters. The microbial biomass carbon in the soil was estimated using the chloroform fumigation extraction method using the formula Bc = Fc/Kc, where Bc represents biomass carbon, Fc represents the difference in the amount of carbon that can be extracted from fumigated and non-fumigated soil, and Kc represents the efficiency factor, which is 0.45 (Vance et al., 1987). The soil extract obtained after fumigation extraction for microbial biomass carbon was digested and examined for total nitrogen in order to estimate the amount of microbial biomass nitrogen (Nelson and Sommers, 1982). Dehydrogenase activity was estimated by adopting the methodology given by Casida et al. (1964). It is based on the principle that 2,3,5-triphenyl tetrazolium chloride (TTC), which is used as an electron acceptor, is reduced to triphenyl formazan (TPF), which imparts color. The quantity of TPF in terms of the color intensity formed was measured using a spectrophotometer at 485 nm wavelength. The acid and alkaline phosphatase activity was carried out by adopting the methodology outlined by Tabatabai and Bremer (1969). It is based on the incubation of soil samples mixed with a buffer solution of p-nitrophenylphosphate at 37°C for 1 h. The released p-nitrophenol is stained and measured spectrophotometrically at 400 nm.

### 2.5 Nutrient use efficiency

The nutrient (N, P, and K) use efficiency parameters, viz., apparent recovery efficiency (RE), agronomic nutrient use efficiency (AE), partial nutrient budget (PNB), utilization efficacy (UE), and partial factor productivity (PFP), were calculated using the following formulae, as per Singh et al. (2021) to know the crop response to the added fertilizers and to compare the use efficiency of added nutrients and economics under different approaches of fertilizer recommendation.


$$\text{ARE}\left(kɡ\;k{ɡ}^{-1}\right)\;=\frac{\text{[Total nutrient uptake in treated plot }\left({\text{kg ha}}^{-1}\right)-\text{Total nutrient uptake in control plot }\left({\text{kg ha}}^{-1}\right)]}{\text{Fertilizer nutrient applied }\left({\text{kg ha}}^{-1}\right)}$$

$$\text{AE}\left(kɡ\;k{ɡ}^{-1}\right)\text{ =}\frac{\text{[Yield in treated plot }\left({\text{kg ha}}^{-1}\right)-\text{Yield in control plot }\left({\text{kg ha}}^{-1}\right)]}{\text{Fertilizer nutrient applied }\left({\text{kg ha}}^{-1}\right)}$$

$$\text{PNB}\left(kɡ\;k{ɡ}^{-1}\right)=\frac{\text{Nutrient uptake }\left({\text{kg ha}}^{-1}\right)}{\text{Fertilizer nutrient applied }\left({\text{kg ha}}^{-1}\right)}$$

$$\text{UE}\left(kɡ\;k{ɡ}^{-1}\right)=\frac{\text{Nutrient uptake by economic part }\left({\text{kg ha}}^{-1}\right)}{\text{Economic Yield }\left({\text{kg ha}}^{-1}\right)}$$

$$\text{PFP}\left(q\;k{ɡ}^{-1}\right)\text{ =}\frac{\text{ Yield }\left({\text{q ha}}^{-1}\right)}{\text{Fertilizer nutrient added }\left({\text{kg ha}}^{-1}\right)}$$

### 2.6 Soil quality index

The tool in question was crafted through a tripartite methodology, initiating with the selection of the minimal dataset (MDS) and culminating in the amalgamation of indicator scores into a soil quality index, as delineated by Andrews et al. (2021). A univariate statistical analysis alongside a correlation matrix of indicators distilled the dataset to its MDS core. Noteworthy variables from different soil parameters, demonstrating significance (*p* < 0.05), were incorporated into the MDS and subjected to principal component analysis (PCA). Utilizing the SPSS software and varimax rotation, PCA was conducted on each pivotal indicator to categorize them into principal component (PC) factors for relational assessment. PCs, boasting an eigenvalue exceeding 1, as per Brejda et al. (2000), and accounting for a minimum of 5% variance in data, were earmarked for indicator selection. Within each PC, the indicator with the most substantial factor loading, be it positive or negative, was scored. To curtail data overlap, multivariate correlation was employed for factors consolidated under a single PC. Variables with a high correlation (>0.60), deemed redundant by Legaz et al. (2017), were narrowed down to a singular representative for the MDS, while the remainder were excised from the dataset. Conversely, uncorrelated yet heavily weighted variables were retained, underscoring their significance to the MDS.

For the computation of the soil quality index (SQI), the top 15 cm of soil underwent analysis for its physical, chemical, and biological characteristics. The selection criteria for the MDS included indicators within 10% of the highest weighted loading for each principal component. Legaz et al. (2017) assessed the influence of each variable in a multifaceted principal component analysis, retaining those with a correlation coefficient of less than 0.60. Each observation of the MDS indicators was standardized. This standardized value is termed the “indicator score” (S). Indicators are assessed using a linear scoring approach and are classified into three categories: “more is better,” “less is better,” and “optimum is better.” In the case of “more is better,” an observation is divided by the maximum observed value, assigning a score of 1 to the highest observation and less than 1 to all others. Conversely, for “less is better,” the minimum observed value is divided by each observation, giving the lowest value indicator a score of 1 and less than 1 to the remaining, until the threshold level is reached. Beyond this point, the scoring for “optimum is better” switches from “more is better” to “less is better.”


(1) L (Y) = X/Xmax   “More is better” approach

(2) L (Y) = Xmin/X   “Less is better” approach

Where,

L(Y) is the linear score varying from 0 to 1,

X is the soil indicator value,

Xmax is the maximum value of each soil indicator, and

Xmin is the minimum value of each soil indicator.

The SQI is computed by integrating the score and weight factor of each indicator. This can be explained by the following equation:


$$SQI=\sum_{i=1}^{n}\text{WiSi}$$

Where,

Si = score for subscripted variable, and

Wi = weighing factor derived from the PCA.

### 2.7 Statistical analysis

The data were subjected to analysis of variance (ANOVA) technique of randomized block design as per the procedure outlined by Gomez and Gomez (1984). Online statistical program OPSTAT was used for the data analysis and least significance difference (LSD) values at *α* = 0.05 were used to compare treatment means. Pearson’s correlation coefficients were used as a measure of the strength of linear dependence between studied parameters at *p* ≤ 0.05 and *p* ≤ 0.01. The SPSS 29.0 software also performed the MDS through PCA for SQI selection. PCA was applied to the correlation matrix of the soil variables in order to obtain a few new components explaining most of the variation of the original variables. The principal components (PCs) that explained cumulatively a high percentage of the total variance and had an eigenvalue greater than one (Kaiser criterion) were retained. Together with the eigenvalue, the percentage of variation explained by the single component was taken into account, considering the threshold of 5% suggested by Wander and Bollero (1999). Variable loadings were examined. Within each PC, only highly weighted loadings, defined as having absolute values within 10% of the highest loading (Sharma et al., 2005), were considered and signs were examined to investigate relationships among selected variables.

## 3 Results

### 3.1 Crop yield

**Table 1** provides a comparative analysis of agricultural yields for various crops under different treatments over the period 2017 to 2022. The STCR NPK + FYM–yield level 1 treatment consistently achieved higher yield increases compared to the general recommended dose, with increases of 8.88% for soybean, 25.85% for sunflower, 16.65% for dry chili, 24.53% for aerobic rice, 22.35% for foxtail millet, 28.70% for okra, and 24.33% for kodo millet. Similarly, the STCR NPK + FYM approach registered higher yields at both yield levels compared to the sole NPK application. The absolute control treatment, which represents the baseline yield without any fertilization, had the lowest yield across all crops, emphasizing the importance of fertilization in crop yield enhancement.

**Table 1:** Effect of soil test crop response alongside various fertilization strategies on the yield and value cost ratio of diverse crops.

Treatments	Soybean (2017)		Sunflower (2018)		Dry chili (2019)		Aerobic rice (2020)		Foxtail millet (2021)		Okra (2022)		Kodo millet (2023)	
	Yield (t ha^–1^)	VCR	Yield (t ha^–1^)	VCR	Yield (t ha^–1^)	VCR	Yield (t ha^–1^)	VCR	Yield (t ha^–1^)	VCR	Yield (t ha^–1^)	VCR	Yield (t ha^–1^)	VCR
STCR NPK–yield level 1	2.18	10.32	2.518	14.91	1.695	12.40	6.761	8.88	1.547	4.05	25.95	32.06	1.67	15.03
STCR NPK + FYM–yield level 1	2.39	5.62	2.713	3.88	1.667	3.40	6.546	10.10	1.407	3.00	26.82	16.25	1.71	4.06
STCR NPK–yield level 2	1.83	9.51	2.426	13.30	1.359	17.84	6.002	9.78	1.371	3.70	22.29	27.36	1.407	14.96
STCR NPK + FYM–yield level 2	2.01	4.30	2.494	6.74	1.447	2.83	5.759	10.99	1.296	2.67	22.84	12.05	1.36	3.02
General recommended dose	2.20	3.31	2.155	2.30	1.429	2.43	5.256	1.57	1.15	0.96	20.84	8.87	1.377	2.78
Soil fertility rating	2.16	3.07	2.299	2.40	1.582	2.97	5.011	1.34	1.226	2.06	21.51	9.27	1.394	2.83
Absolute control	1.47		1.052		0.56		2.068		0.985		13.89		0.527	
**SEm±**	**0.068**		**0.066**		**0.066**		**0.20**		**0.096**		**0.074**		**0.300**	
**CD @ 5%**	**0.193**		**0.187**		**0.188**		**0.562**		**0.272**		**0.211**		**0.086**	

SEm, Standard error of Mean; CD, Critical Difference.

### 3.2 Nutrient uptake

The data on nutrient uptake as influenced by different approaches of fertilizer recommendation are depicted in **Table 2**. Significantly higher uptake of NPK among all the crops, viz., soybean (95.78, 28.95, and 69.67 kg NPK ha^−1^), sunflower (65.50, 43.95, and 79.67 kg NPK ha^−1^), dry chili (72.38, 7.17, and 56.30 kg NPK ha^−1^), aerobic rice (84.00, 13.82, and 124.25 kg NPK ha^−1^), foxtail millet (54.33, 22.17, and 68.32 kg NPK ha^−1^), okra (150.93, 54.62, and 160.396 kg NPK ha^−1^), and kodo millet (22.61, 7.6, and 71.71 kg NPK ha^−1^) was recorded with the application of fertilizer based on the STCR approach along with FYM at yield level 1 compared to the other treatments. The treatment with absolute control invariably resulted in minimal absorption of nutrients among all the crops tested.

**Table 2:** Effect of soil test crop response alongside various fertilization strategies on the NPK uptake in different crops.

Treatments	Soybean			Sunflower			Dry chili			Aerobic rice			Foxtail millet			Okra			Kodo millet		
	N	P	K	N	P	K	N	P	K	N	P	K	N	P	K	N	P	K	N	P	K
	kg ha^–1^																				
STCR NPK–yield level 1	92.62	27.25	60.05	54.97	42.25	70.05	74.93	7.11	50.25	89.38	14.85	110.95	58.19	23.40	70.15	137.70	50.19	150.13	21.32	6.82	69.79
STCR NPK + FYM–yield level 1	95.78	28.95	69.67	62.50	43.95	79.67	72.38	7.17	56.30	84.00	13.82	124.25	54.33	22.17	68.32	150.93	54.62	160.39	22.61	7.61	71.71
STCR NPK–yield level 2	78.23	26.24	53.43	49.03	41.24	63.43	63.35	5.83	38.58	82.02	13.74	102.67	53.12	21.33	67.12	113.16	37.56	120.73	21.61	5.21	60.17
STCR NPK + FYM–yield level 2	82.90	26.22	56.16	52.92	41.22	66.16	62.74	6.27	46.06	77.27	13.26	117.22	50.70	19.91	63.90	124.41	43.78	131.37	18.87	4.72	59.86
General recommended dose	83.93	23.09	49.63	53.20	38.09	59.63	62.45	5.49	38.48	70.41	11.88	105.89	44.58	16.64	56.94	111.56	39.08	107.40	19.20	6.44	57.89
Soil fertility rating	82.26	23.71	50.42	51.99	38.71	60.42	62.27	5.52	35.97	62.37	11.07	95.65	47.77	17.99	57.38	125.43	54.05	122.75	21.18	6.10	62.03
Absolute control	62.57	11.25	29.10	20.05	26.25	39.10	23.13	2.54	15.92	25.48	4.91	36.96	35.97	12.89	44.18	94.32	32.41	84.84	6.44	2.15	21.36
**SEm±**	**4.02**	**2.18**	**3.12**	**1.42**	**2.20**	**3.12**	**5.50**	**0.72**	**6.16**	**6.22**	**0.74**	**9.50**	**2.54**	**1.81**	**4.18**	**1.43**	**1.84**	**2.37**	**0.60**	**0.43**	**3.04**
**CD @ 5%**	**11.45**	**6.20**	**8.88**	**4.06**	**6.60**	**8.88**	**15.67**	**2.04**	**17.55**	**17.73**	**2.11**	**27.07**	**7.83**	**2.56**	**12.88**	**4.48**	**5.75**	**7.39**	**1.70**	**1.24**	**8.66**

SEm, Standard error of Mean; CD, Critical Difference.

### 3.3 Nutrient use efficiency

The highest apparent recovery efficiency of nitrogen and potassium was observed in STCR NPK + FYM at yield level 2 with absorption rates of 0.73 and 4.98 kg kg^−1^ (**Table 3**). Similarly, the recovery efficiency of phosphorus was higher in STCR NPK + FYM at yield level 1. However, minimal apparent recovery efficiency (ARE) for nitrogen was observed in the soil fertility rating approach, registering at 0.34 kg kg^−1^. Regarding phosphorus (P), the STCR NPK–YIELD Level 2 treatment exhibits the least ARE at 0.17 kg per kg. For potassium (K), both the general recommended dose and soil fertility rating treatments show the lowest ARE, with values of 0.90 and 0.86 kg kg^−1^, respectively. The agronomic nutrient use efficiency (ANUE) for nitrogen, phosphorus, and potassium in STCR NPK + FYM at yield levels 2 and 1 shows significant increases over the general recommended doses. The ANUE of N increased by 125%, P by 51.54%, and K by 187.17%, under STCR NPK + FYM at yield level 1 compared to the general recommended dose, indicating a substantial improvement in nutrient utilization with the STCR NPK + FYM approach.

**Table 3:** Effect of soil test crop response alongside various fertilization strategies on nutrient use efficiency.

Treatments	ARE			ANUE			PNB			UE			PFP		
	N	P	K	N	P	K	N	P	K	N	P	K	N	P	K
	kg kg^−1^										kg q^−1^				
STCR NPK–yield level 1	0.42	0.19	1.51	27.50	33.01	45.12	1.13	0.36	2.38	0.41	1.79	0.32	42.26	49.48	68.11
STCR NPK + FYM–yield level 1	0.67	0.26	3.91	60.93	45.66	102.98	2.79	0.49	5.94	0.41	1.85	0.30	87.11	67.45	151.81
STCR NPK–yield level 2	0.42	0.17	1.34	29.52	33.28	43.80	1.32	0.39	2.52	0.39	1.86	0.32	45.87	53.47	70.65
STCR NPK + FYM–yield level 2	0.73	0.24	4.98	54.35	48.60	130.16	2.59	0.54	8.59	0.41	1.94	0.31	81.10	77.25	210.82
General recommended dose	0.43	0.18	0.90	27.00	30.13	35.86	1.22	0.38	1.74	0.43	1.82	0.36	46.64	50.33	61.26
Soil fertility rating	0.34	0.25	0.86	23.28	41.16	35.35	0.99	0.51	1.55	0.45	1.95	0.39	39.40	66.80	58.65
Absolute control	–	–	–	–	–	–	–	–	–	0.41	1.58	0.31	–	–	–

ARE, apparent recovery efficiency; ANUE, agronomic nutrient use efficiency; PNB, partial nutrient budget; UE, utilization efficiency; PFP, partial factor productivity.

The study reveals that combining STCR NPK + FYM at yield levels 1 and 2 improved PNB for NPK. The most notable increase was seen in nitrogen at yield level 1 (2.79), and phosphorus and potassium were higher at yield level 2 (0.54 and 8.59, respectively). On the other hand, UE was higher under the general recommended dose and soil fertility rating approach. However, the values were comparable with the STCR approach of fertilizer recommendation. Similarly, the partial factor productivity was higher for nitrogen in the treatment STCR NPK + FYM at yield level 1 (87.11 kg q^−1^), and the PFP for phosphorus (77.25 kg q^−1^) and potassium (210.82 kg q^−1^) was the highest in STCR NPK + FYM at yield level 2.

### 3.4 Soil properties

The soil fertility rating treatment exhibited the highest pH value at 5.89, marking a 4.25% increase over the absolute control baseline of 5.65 (**Table 4**). Conversely, the absolute control also recorded the lowest electrical conductivity (EC) at 0.09 dS m^−1^, which is a significant 57.14% lower than the highest EC observed in STCR NPK–yield level 1 at 0.21 dS m^−1^. Additionally, the STCR NPK + FYM–yield level 1 treatment showed the highest levels of organic carbon (OC), nitrogen (N), and potassium (K), at 0.5%, 288.58 kg ha^–1^, and 156.72 kg ha^–1^, respectively. These figures represent increases of 1.34-fold for OC, 1.27-fold for N, and 1.55-fold for K when compared to the absolute control values of 0.35% OC, 120.56 kg N ha^–1^, and 96.28 kg K ha^–1^. However, the general recommended dose has the highest available phosphorous content at 150.28 kg ha^−1^.

**Table 4:** Effect of soil test crop response alongside various fertilization strategies on the soil properties.

Treatments	pH	Electrical conductivity	Organic carbon	Avail. N	Avail. P_2_O_5_	Avail. K_2_O
	(1:2.5)	dS m^−1^	%	kg ha^–1^		
STCR NPK–yield level 1	5.66	0.21	0.43	281.56	121.25	145.56
STCR NPK + FYM–yield level 1	5.81	0.20	0.50	288.58	130.56	156.72
STCR NPK–yield level 2	5.73	0.19	0.4	230.58	114.58	125.58
STCR NPK + FYM–yield level 2	5.79	0.2	0.46	245.49	129.56	141.58
General recommended dose	5.68	0.18	0.45	254.56	150.28	115.25
Soil fertility rating	5.89	0.17	0.41	270.56	95.68	119.71
Absolute control	5.65	0.09	0.35	120.56	55.69	96.28
**SEm±**	**0.09**	**0.01**	**0.01**	**6.5**	**12.49**	**8.62**
**CD @ 5%**	**0.26**	**0.02**	**0.02**	**18.53**	**35.6**	**24.57**

SEm, Standard error of Mean; CD, Critical Difference.

### 3.5 Principal component analysis

**Figure 2:**
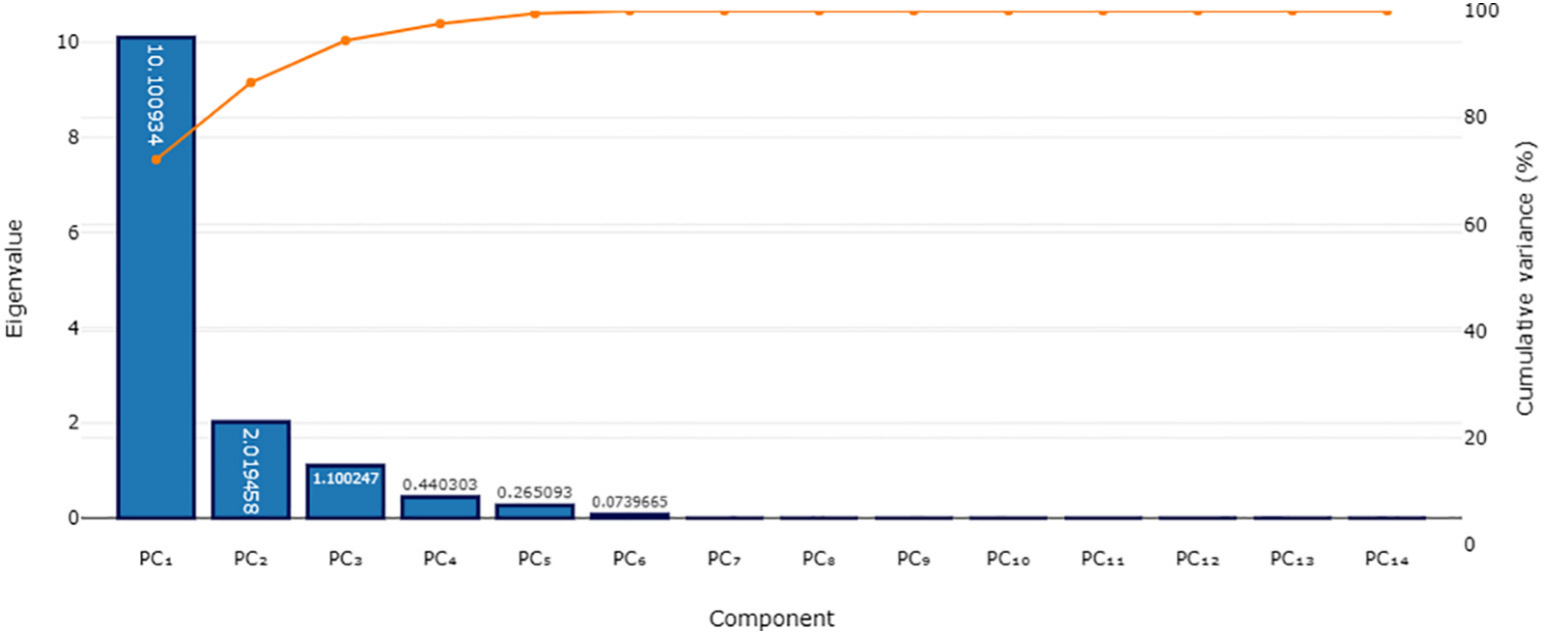
Scree plot explaining the relationship of the eigenvalues and the principal components.

Significant correlations (*p* < 0.05) among most soil properties led to their inclusion in the PCA for MDS development (**Table 5**). Only principal components with eigenvalues greater than 1 were considered, as depicted in **Figure 2**. The PCA revealed that the first two components accounted for 86.57% of the data variance, with PC1 and PC2 individually explaining 72.15% and 14.42%, respectively (**Table 6**; **Figure 2**). Factors such as dehydrogenase activity, organic carbon, electrical conductivity, nitrogen, potassium, acid phosphatase, microbial biomass carbon, and nitrogen received the highest loadings in PC1 and were highly intercorrelated, leading to the selection of dehydrogenase activity for the MDS. Bulk density, which had a higher loading in PC2 as shown in **Table 7**, was also included. Indicators were normalized on a 0 to 1 scale using a linear scoring function, with weighted factors of 0.83 for PC1 and 0.17 for PC2, culminating in the formulation of the SQI equation.

**Table 5:** Pearson correlation coefficient values (*r*) between various soil quality attributes of 0–15 cm soil layer.

p\Group	BD	MWHC	Porosity	Ph	EC	OC	N	P	K	MBC	MBN	DHA	AP	ALP
**BD**	1													
**MWHC**	−0.55	1												
**Porosity**	−0.53	1	1											
**pH**	−0.12	0.41	0.41	1										
**EC**	−0.15	0.82	0.83	0.033	1									
**OC**	−0.35	0.8	0.8	0.12	0.84	1								
**N**	−0.12	0.81	0.82	0.23	0.95	0.91	1							
**P**	−0.14	0.74	0.75	−0.12	0.95	0.71	0.83	1						
**K**	0.086	0.59	0.6	−0.056	0.87	0.8	0.88	0.73	1					
**MBC**	0.037	0.67	0.68	−0.064	0.93	0.76	0.87	0.83	0.97	1				
**MBN**	0.036	0.67	0.68	−0.064	0.93	0.76	0.87	0.83	0.97	1	1			
**DHA**	0.04	0.71	0.72	0.014	0.93	0.77	0.89	0.83	0.96	0.99	0.99	1		
**AP**	0.1	0.62	0.64	−0.13	0.91	0.76	0.85	0.83	0.96	0.99	0.99	0.99	1	
**ALP**	0.083	0.65	0.66	0.28	0.73	0.74	0.8	0.54	0.88	0.86	0.86	0.89	0.86	1

MWHC, Minimum water holding capacity; EC, Electrical Conductivity; OC, Organic Carbon; N, Nitrogen; P, Phosphorus; K, Potassium; MBC, Microbial Biomass Carbon; MBN, Microbial Biomass Nitrogen; DHA, Dehydrogenase; AP, Acid Phosphatase; ALP, Alkaline Phosphatase.

**Table 6:** Eigenvalues from principal component analysis (PCA) of soil quality parameters.

Principal component	Initial eigenvalues			Weightage factor
	Total	% Variance	Cumulative %	
1	10.10	72.15	72.15	0.83
2	2.02	14.42	86.57	0.17
			**Total**	1.00

**Table 7:** Principal component analysis of soil quality parameters.

Component matrix		
	PC1	PC2
Dehydrogenase activity	**0.972**	0.197
Electrical conductivity	**0.971**	−0.004
Microbial biomass carbon	**0.961**	0.242
Microbial biomass nitrogen	**0.961**	0.242
Nitrogen	**0.955**	−0.083
Acid phosphatase	**0.944**	0.312
Available potassium	**0.929**	0.289
Organic carbon	**0.881**	−0.206
Available phosphorus	0.871	0.026
Alkaline phosphatase	0.862	0.102
Porosity	0.839	−0.526
Maximum water holding capacity	0.830	−0.541
Bulk density	−0.139	**0.812**
pH	0.100	−0.627
**10%HF**	**0.097201**	**0.081248**
	**0.875**	**0.731**

Boldface eigenvalues correspond to the PCs examined for the index. Boldface factor loadings are considered highly weighed; Bold-underlined factors correspond to the indicators included in the index.

PC, principal component.


SQI=0.83×DHA score+0.17×bulk density score (2)

The STCR NPK + FYM plots under yield level 1 registered a high DHA and low bulk density. The nutrient management options clearly manifested their effect on these two soil parameters, making these apt SQI indicators. The SQI was significantly higher under the STCR NPK + FYM–yield level 1 (0.98) treatment as compared to the other treatments (**Table 8**). Furthermore, the hierarchy of fertilizer recommendation approaches on SQI was as follows: STCR NPK + FYM–yield level 1 > STCR NPK + FYM–yield level 1 > STCR NPK–yield level 1 > STCR NPK–yield level 1 > soil fertility rating > general recommendation dose > absolute control.

**Table 8:** Score, weight, and soil quality index values of selected minimum dataset variables under STCR alongside various fertilization strategies in different crops.

Treatments	Dehydrogenase activity			Bulk density			SQI
	S	W	T	S	W	T	
STCR NPK–yield level 1	0.93	0.83	**0.78**	0.925	0.17	**0.16**	0.94
STCR NPK + FYM–yield level 1	0.98	0.83	**0.83**	0.890	0.17	**0.15**	0.98
STCR NPK–yield level 2	0.92	0.83	**0.77**	0.932	0.17	**0.16**	0.93
STCR NPK + FYM–yield level 2	1.00	0.83	**0.82**	0.897	0.17	**0.15**	0.97
General recommended dose	0.8	0.83	**0.70**	0.932	0.17	**0.16**	0.86
Soil fertility rating	0.88	0.83	**0.74**	0.937	0.17	**0.16**	0.90
Absolute control	0.65	0.83	**0.55**	1.000	0.17	**0.17**	0.72

W, weightage factor; S, score value; T, total; SQI, soil quality index.

**Figure 3:**
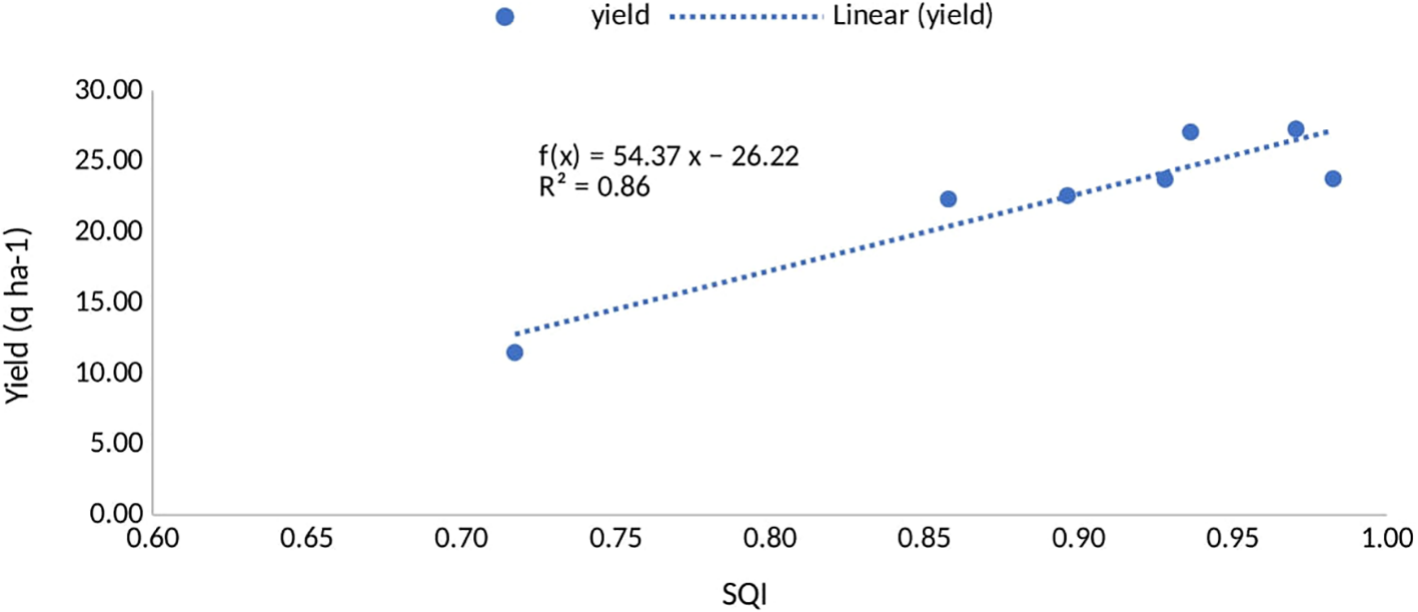
Relationship between soil quality index with crop yield under different fertilizer recommendation approaches.

In the study, plots treated with STCR NPK + FYM at yield levels 1 and 2 exhibited SQI values that were 13.95% and 12.79% greater, respectively, than those treated with the general recommendation dose, as shown in **Table 8**. The established SQI was corroborated by the mean yield of seven different crops, as depicted in **Figure 3**, which presented a scattered plot and a linear trend line with an *R* ^2^ value of 0.85.

## 4 Discussion

The integration of FYM with NPK fertilization based on the STCR approach appears to be beneficial for most crops at yield level 1, with some exceptions at yield level 2, particularly for aerobic rice and foxtail millet where the sole use of NPK seemed more effective. The data suggest that the combined use of organic and inorganic fertilizers can enhance crop yields, aligning with sustainable agricultural practices which might be due to enhanced microbial activity and conversion of unavailable nutrients into available forms and also due to improved physical, chemical, and biological properties (Katkar et al., 2011) that lead to increased productivity. Similarly, the higher yield under the STCR NPK + FYM approach might be due to the balanced application of nutrients which is based on soil analysis and considers the amount of nutrients removed by the crops, the initial levels of soil fertility, the efficiency of nutrients present in the soil, manure added through the fertilizers (Krishna Murthy et al., 2023a), and the ability of targeted yield approaches to satisfy the nutrient demand of crop more efficiently. These factors might have provided the optimum nutrients at the optimum time for better uptake and ultimately resulted in higher dry matter and yield (Krishna Murthy et al., 2023b).

The STCR NPK + FYM treatment generally resulted in higher nutrient uptake (NPK) across most crops and other fertilizer recommendation approaches, except dry chili (N) and aerobic rice (N, P), where STCR NPK alone had higher uptake (**Table 2**). This suggests that the combined use of organic and inorganic fertilizers can enhance nutrient absorption by crops, which is beneficial for crop growth and yield. Specifically, dry chili demonstrated the least assimilation of phosphorus and potassium across the majority of treatments, suggesting that a specialized fertilization approach may be necessary for this particular crop (Singh et al., 2021).

The data indicated in **Table 3** show that the treatment STCR NPK + FYM–yield level 1 generally had the highest nutrient use efficiency, suggesting that it may be the most effective treatment among those listed. Combining STCR with FYM seems to boost nutrient recovery. FYM improves soil quality and nutrient availability, enhancing the absorption of nutrients by crops thereby increasing crop productivity (Abid et al., 2020). Conversely, the general recommended dose and soil fertility rating treatments tend to show the lowest values, indicating they may be less effective compared to the other treatments due to the soil’s inherent limitations in providing nitrogen without supplemental fertilization (Barłóg et al., 2022). The least recovery of phosphorus in the soil fertility rating approach was potentially due to the ineffectiveness of the nutrient management approach that excludes FYM in rendering phosphorus accessible to plants. Similarly, lower potassium recovery in the general recommended dose and soil fertility rating indicates that standard guidelines and soil fertility assessments may fall short in ensuring adequate potassium uptake, likely a consequence of potassium’s reduced solubility under these conditions (Andrews et al., 2021). These findings highlight the importance of integrating organic amendments like FYM with tailored nutrient management strategies to maximize nutrient recovery efficiency, particularly for potassium and nitrogen.

The most notable improvements of ANUE were observed at yield level 1 for nitrogen and yield level 2 for phosphorus and potassium, suggesting that FYM may improve soil fertility and structure (Meena et al., 2018). In contrast, the general recommended doses showed lower ANUE, implying that customized STCR NPK + FYM treatments could be more effective for nutrient utilization (Rao et al., 2021). The balance of nutrients available to the crop can affect the uptake of N, P, and K, as they can interact synergistically or antagonistically (Xie et al., 2021). These findings are essential for understanding how to optimize nutrient use for maximum crop production while ensuring environmental sustainability. By analyzing PFP, farmers and agronomists can make informed decisions about fertilizer application rates and methods (Huang and Karimanzira, 2018).

Based on the data, adding FYM to STCR NPK significantly increases the organic carbon content and the availability of nitrogen and potassium (Moharana et al., 2017). The application of STCR NPK + FYM in yield level 1 plots resulted in elevated DHA and reduced bulk density, establishing them as suitable indicators of soil quality (Sharma et al., 2019). These indicators, reflecting the soil’s physical and chemical attributes, showed a strong correlation with other soil parameters across physical, chemical, and biological spectrums (Parihar et al., 2020). The findings indicated that the STCR NPK + FYM treatment markedly improved soil quality indicators, with yield level 1 plots exhibiting an SQI that was 13.95% greater than that achieved with the general recommendation dose. STCR-based treatments reported higher SQI when compared with RDF due to judicious, balanced, and profitable fertilization along with the use of organic manure in a definite proportion based on nutrient status of the soil and as required by the crops (Sharma et al., 2016). Application of fertilizers and manures based on the STCR approach not only helps in achieving the target yield but also maintains overall soil health (Sharma et al. 2019). Among the STCR-based treatments, the combined application of inorganic fertilizers and FYM resulted in better soil quality when compared with the use of inorganic fertilizers alone (Huang et al., 2010). The synergistic approach of combining organic and inorganic nutrients, along with strategic management, not only bolstered soil physical health but also maximized nutrient efficiency, which in turn was linked to an increase in biomass production, corroborated by studies from Sapkota et al. (2014) and Parihar et al. (2017).

## 5 Conclusion

The findings of this study suggest that the STCR NPK + FYM approach could be a viable strategy for improving soil health and achieving yield sustainability in Alfisols of southern India. The percent achievement of the targeted yield was within ±10% variance at both targets, demonstrating the validity of the equations for prescribing integrated fertilizer doses. The increased crop yields, improved nutrient uptake, and enhanced soil quality indicators provide a strong foundation for recommending this balanced fertilization method as a cornerstone for sustainable agricultural practices. Continuous research and development activities to refine STCR models for different crops and regions will ensure accuracy and effectiveness. Also, integrating STCR with advanced technologies like GPS, GIS, and remote sensing can enhance precision in fertilizer application.

## Data Availability

The datasets presented in this study can be found in online repositories. The names of the repository/repositories and accession number(s) can be found in the article/**Supplementary Material**.
