# Supplementary material for: Soil test crop response nutrient prescription equations for improving soil health and yield sustainability—a long-term study under Alfisols of southern India
Source: Front Plant Sci. 2024 Nov 13;15:1439523. doi: 10.3389/fpls.2024.1439523 (PMC11601127; doi:10.3389/fpls.2024.1439523)
Supplement: Supplementary file 1 [file DataSheet1.pdf]

Supplementary Material

Supplementary Material 1: List of Equations developed and validated from 2017-2023 for different crops at AICRP on STCR, Bangalore Center

SI. No	Crop	Genetic potential yield	Equation validation year	Targeted Yield Equations
1	Sunflower	2.0-2.2 t ha^{-1}	2017	FN = 8.38 T – 0.57 STV<br>FP_2O_5 = 8.05 T – 6.00 STV<br>FK_2O = 9.87 T – 0.47 STV
2	Soybean	2.5-3.0 t ha^{-1}	2018	FN = 3.50 T – 0.27 STV - 0.0031 OM<br>FP_2O_5 = 7.48 T – 0.50 STV-0.0067 OM<br>FK_2O = 7.20 T – 0.46 STV-0.0045 OM
3	Dry chilli	1.2-1.5 t ha^{-1}	2019	FN = 20.38 T - 0.35 STV - 0.000525 OM<br>FP_2O_5 = 8.26 T – 0.60 STV - 0.000816 OM<br>FK_2O = 8.58 T - 0.24 STV - 0.000399 OM
5	Aerobic rice	5.5-6.5 t ha^{-1}	2021	Inorganic<br>FN = 3.03 T – 0.20 STV<br>FP_2O_5 = 1.25 T – 0.07 STV<br>FK_2O = 1.51 T – 0.23 STV<br>Integrated<br>FN = 2.89 T – 0.20 STV – 0.73 OM<br>FP_2O_5 = 1.13 T – 0.07 STV – 0.49 OM<br>FK_2O = 1.50 T – 0.21 STV – 0.42OM
4	Foxtail millet	1.2-1.5 t ha^{-1}	2020	Inorganic<br>FN = 7.19 T – 0.26 STV<br>FP_2O_5 = 2.68 T – 0.068 STV<br>FK_2O = 3.12 T – 0.102 STV<br>Integrated<br>FN = 5.80 T – 0.219 STV – 0.49 OM<br>FP_2O_5 = 2.38 T – 0.055 STV – 0.32 OM<br>FK_2O = 2.07 T – 0.064 STV – 0.93 OM
6	Okra	20-25 t ha^{-1}	2022	Inorganic<br>FN = 1.37 T – 0.39 STV<br>FP_2O_5 = 0.57 T – 0.15 STV<br>FK_2O = 0.60 T – 0.24 STV<br>Integrated<br>FN = 1.14 T – 0.33 STV –0.67 OM<br>FP_2O_5 = 0.52 T – 0.12 STV – 0.62 OM<br>FK_2O = 0.56 T – 0.23 STV – 0.86 OM
7	Kodo millet	1.2-1.5 t ha^{-1}	2023	Inorganic<br>FN = 3.79 T – 0.20 STV<br>FP_2O_5 = 2.65 T – 0.36 STV<br>FK_2O = 2.76 T –0.31 STV<br>Integrated<br>FN = 4.00 T – 0.21 STV - 0.32 OM<br>FP_2O_5 = 2.38 T – 0.66 STV- 0.19 OM<br>FK_2O = 1.80 T–0.16 STV–0.77 OM

Supplementary Material 2: Initial NPK Soil Test Readings and Fertilizer Quantities for Crop Trials (2017-2023)

Soybean						
**Treatments**	**Initial soil test values (kg ha^{-1})**			**Fertilizer dose (kg ha^{-1})**		
	**N**	**P**	**K**	**N**	**P**	**K**
STCR NPK- Yield Level 1	235.25	67.85	180.25	41.95	120.00	56.25
STCR NPK + FYM - Yield Level 1	250.25	68.58	183.58	7.35	59.26	62.36
STCR NPK- Yield Level 2	231.58	66.89	190.58	25.44	120.00	56.25
STCR NPK + FYM - Yield Level 2	255.56	66.58	181.58	7.50	44.70	26.17
General recommended dose	240.15	62.58	194.52	30.00	80.00	37.50
Soil fertility rating	250.14	60.25	190.52	37.50	60.00	37.50
Absolute control	230.25	59.66	189.25	0.00	0.00	0.00
**Sunflower**						
**Treatments**	**Initial soil test values (kg ha^{-1})**			**Fertilizer dose (kg ha^{-1})**		
	**N**	**P**	**K**	**N**	**P**	**K**
STCR NPK- Yield Level 1	229.60	92.55	182.00	29.00	0.00	40.00
STCR NPK + FYM - Yield Level 1	246.47	90.71	196.40	29.00	0.00	40.00
STCR NPK- Yield Level 2	227.88	93.54	150.00	32.00	0.00	35.00
STCR NPK + FYM - Yield Level 2	248.87	81.43	176.80	32.00	0.00	35.00
General recommended dose	236.69	68.84	142.00	37.50	50.00	37.50
Soil fertility rating	231.56	68.30	151.60	46.88	50.00	46.88
Absolute control	220.56	42.73	110.00	0.00	0.00	0.00
**Dry Chilli**						
**Treatments**	**Initial soil test values (kg ha^{-1})**			**Fertilizer dose (kg ha^{-1})**		
	**N**	**P**	**K**	**N**	**P**	**K**
STCR NPK- Yield Level 1	233.56	55.01	162.08	182	75	73
STCR NPK + FYM - Yield Level 1	241.89	45.22	170.5	179	80	107
STCR NPK- Yield Level 2	231.61	60.57	132.4	122	46	54
STCR NPK + FYM - Yield Level 2	236.19	69.97	150.76	120	41	50
General recommended dose	235.53	58.45	111.8	100	50	50
Soil fertility rating	238.93	54.08	144.58	125	50	50
Absolute control	215.89	44.89	80.9	0	0	0
**Aerobic rice**						
**Treatments**	**Initial soil test values (kg ha^{-1})**			**Fertilizer dose (kg ha^{-1})**		
	**N**	**P**	**K**	**N**	**P**	**K**
STCR NPK- Yield Level 1	260.587	75.28	182	144	74	75
STCR NPK + FYM - Yield Level 1	261.667	80.25	196.4	131	61	69
STCR NPK- Yield Level 2	260.213	89.56	150	114	61	61
STCR NPK + FYM - Yield Level 2	262.08	92.58	176.8	102	50	57

General recommended dose	268.05	96.09	142	100	50	50
Soil fertility rating	266.56	98.5497	151.6	125	37.5	50
Absolute control	243.41	56.9604	110	0	0	0
**Foxtail Millet**						
**Treatments**	**Initial soil test values (kg ha^{-1})**			**Fertilizer dose (kg ha^{-1})**		
	**N**	**P**	**K**	**N**	**P**	**K**
STCR NPK- Yield Level 1	275.09	100.81	175.25	49.4992	28.014	22.5766
STCR NPK + FYM - Yield Level 1	289.31	99.18	190.56	39.0395	23.5086	11.7187
STCR NPK- Yield Level 2	265.07	103.24	151.82	29.5223	19.8075	15.6248
STCR NPK + FYM - Yield Level 2	270.15	101.47	185.98	21.707	16.2543	5.78767
General recommended dose	272.13	102.21	145.69	40.00	40.00	0.00
Soil fertility rating	268.95	88.61	150.73	50.00	30.00	0.00
Absolute control	239.12	57.51	115.81	0.00	0.00	0.00
**Okra**						
**Treatments**	**Initial soil test values (kg ha^{-1})**			**Fertilizer dose (kg ha^{-1})**		
	**N**	**P**	**K**	**N**	**P**	**K**
STCR NPK- Yield Level 1	262.25	110.25	130.25	161.55	112.5	118.74
STCR NPK + FYM - Yield Level 1	288.48	119.56	144.58	173.05	100.15	85.25
STCR NPK- Yield Level 2	235.58	102.25	125.89	138.85	110.06	101.79
STCR NPK + FYM - Yield Level 2	261.89	112.50	141.59	147.63	85.4	69.13
General recommended dose	250.17	135.25	115.25	125.00	75.00	62.50
Soil fertility rating	265.48	91.59	119.71	156.25	56.25	78.13
Absolute control	198.56	51.52	96.28	0.00	0.00	0.00
**Kodo Millet**						
**Treatments**	**Initial soil test values (kg ha^{-1})**			**Fertilizer dose (kg ha^{-1})**		
	**N**	**P**	**K**	**N**	**P**	**K**
STCR NPK- Yield Level 1	281.56	121.25	145.56	29.87	18.25	9.53
STCR NPK + FYM - Yield Level 1	288.58	130.56	156.72	21.96	11.75	2.52
STCR NPK- Yield Level 2	230.58	114.58	125.58	28.67	15.3	8.61
STCR NPK + FYM - Yield Level 2	245.49	129.56	141.59	19.58	9.05	1.49
General recommended dose	254.56	150.28	115.25	20.00	20.00	20.00
Soil fertility rating	270.56	95.68	119.71	25.00	15.00	25.00
Absolute control	120.57	55.69	96.28	0.00	0.00	0.00

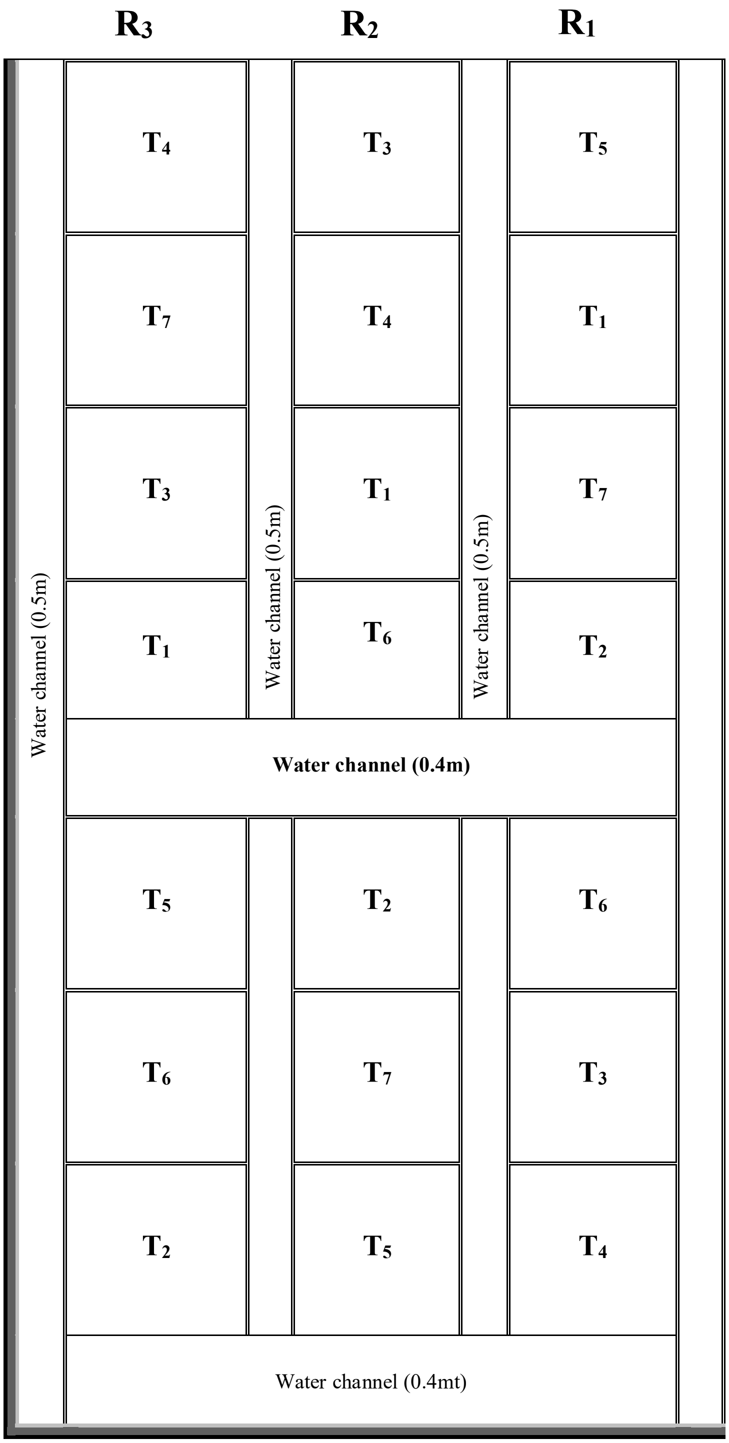

Plot size: 10.8 x 3.0 = 32.40 m^2
Bund spacing: 0.3m

Treatment details

T_1 = STCR NPK for yield level 1

T_2 = STCR NPK with FYM for yield level 1

T_3 = STCR NPK for yield level 2

T_4 = STCR NPK with FYM for yield level 2

T_5 = General recommended dose

T_6 = Soil fertility rating

T_7 = Absolute control

Supplementary Material 3: Layout of the field experiment

a)

b)

c)

d)

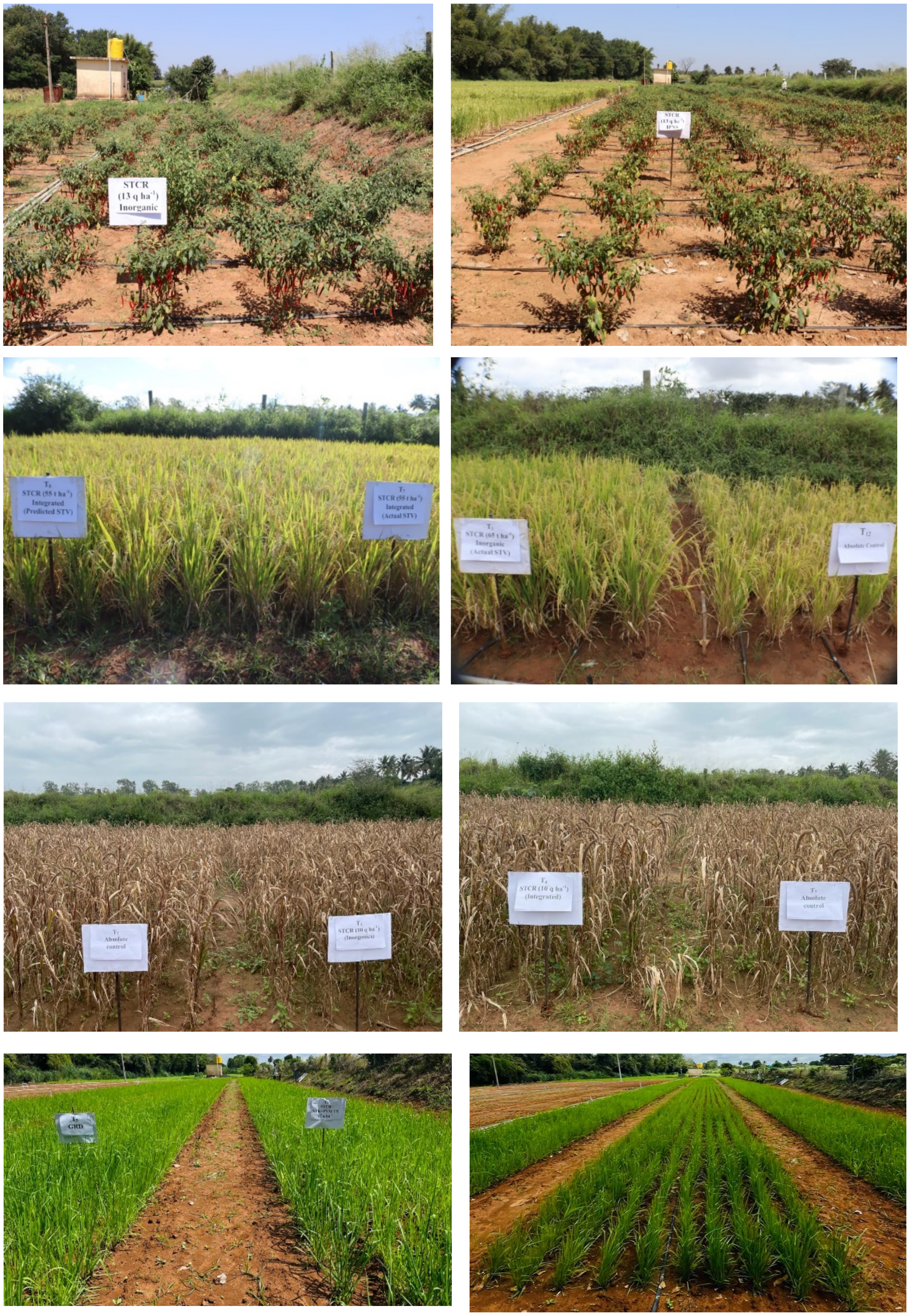

Supplementary Material 3: View of a) Dry chilli b) Aerobic rice c)Foxtail millet and d) kodomillet under the study
